# Artificial intelligence in thoracic surgery consultations: evaluating the concordance between a large language model and expert clinical decisions

**DOI:** 10.3389/fdgth.2025.1633278

**Published:** 2025-11-17

**Authors:** Carlos Déniz, Judith Marcè, Iván Macia, Francisco Rivas, Anna Muñoz, Marina Paradela, Samuel García, Camilo Moreno, Ines Serratosa, Marta García, Tania Rodríguez-Martos, Amaia Ojanguren

**Affiliations:** 1 Department of Thoracic Surgery, Hospital Universitari de Bellvitge, L’Hospitalet de Llobregat, Barcelona, Spain; 2 Bellvitge Institute for Biomedical Research, L’Hospitalet de Llobregat, Barcelona, Spain; 3 Department of Medical Oncology, Catalan Institute of Oncology, L’Hospitalet de Llobregat, Barcelona, Spain; 4 Universitat de Barcelona (UB—Barcelona University), Barcelona, Spain

**Keywords:** artificial intelligence, large language models, thoracic surgery, clinical decision support, oncology, AI in medicine

## Abstract

**Background:**

Artificial intelligence (AI) and large language models (LLMs) are increasingly used in clinical workflows, but their real-world application in thoracic surgery decision-making remains underexplored.

**Methods:**

This retrospective observational study assessed the concordance between diagnostic and therapeutic recommendations generated by Scholar GPT (based on GPT-4) and decisions made by board-certified thoracic surgeons. All outpatient consultations over one week in a tertiary care hospital were included. Each case was evaluated using a 6-point concordance scale (0–5), developed to quantify agreement in diagnosis and treatment planning. This was a retrospective observational, single-centre analysis; two independent thoracic surgeons assigned the concordance score. We report descriptive statistics and used *t*-tests/ANOVA for continuous variables and chi-square tests for categorical variables. Given the exploratory design, no *a priori* sample-size calculation or power analysis was performed.

**Results:**

A total of 81 consultations were analysed. The mean concordance score was 3.67 ± 1.17. High concordance (scores 4–5) occurred in 56.8% of cases, particularly in oncological diagnoses such as mediastinal and pleural tumours. Lower concordance was observed in complex or functional conditions like metastatic lung disease and thoracic outlet syndrome. No significant differences were found between consultation modalities or visit types.

**Conclusion:**

Scholar GPT demonstrated promising alignment with surgeon decisions in structured oncologic cases but showed variability in complex scenarios. While AI may assist in streamlining outpatient workflows, its use should remain complementary to expert clinical judgment. These findings are exploratory and should be interpreted with caution given the small sample size and single-centre, one-week design.

## Introduction

Thoracic surgery involves intricate clinical decision-making that requires the integration of patient history, imaging studies, and adherence to evolving clinical guidelines. The increasing complexity of medical data has driven the exploration of artificial intelligence (AI) and large language models (LLMs) as potential decision-support tools to assist clinicians in managing complex cases with greater efficiency and consistency (1–3). Among these technologies, LLMs such as ChatGPT/Scholar GPT, based on GPT-4-class architectures, have shown strong performance on knowledge-based tasks and standardized examinations, suggesting an emerging role in supporting clinical reasoning (4).

Although early applications of AI in thoracic surgery have focused on imaging and tumor classification (5), there is a growing body of work examining LLMs beyond image-centric tasks in ambulatory clinical decision support—covering diagnostic reasoning, triage, and guideline-concordant recommendations (6–8). However, the application of LLMs in real-world surgical environments—especially in thoracic surgery—remains underexplored, where decisions depend on nuanced presentations, comorbidities, and patient preferences.

Moreover, integrating AI into clinical workflows raises critical questions about its concordance with human judgment and its reliability across heterogeneous scenarios. Prior reports highlight both promising alignment and meaningful discrepancies, underscoring the need for careful validation in contexts where multidisciplinary input remains essential (2, 9, 10). In parallel, potential risks—including hallucinations, misplaced confidence, bias propagation, and over-reliance—carry direct safety implications in low-concordance cases; the “black-box” nature of LLMs further complicates accountability and clinician trust, reinforcing the importance of explainability and human-in-the-loop oversight in high-stakes settings (11–13).

Accordingly, we conducted a single-centre, exploratory evaluation of concordance between Scholar GPT and board-certified thoracic surgeons in routine outpatient consultations, aiming to identify clinical areas of higher and lower agreement and to delineate pragmatic considerations for safe integration.

## Materials and methods

### Study design and population

This retrospective observational study assessed the concordance between AI-generated recommendations from Scholar GPT and clinical decisions made by board-certified thoracic surgeons in a high-volume tertiary university hospital. All outpatient thoracic surgery consultations conducted over one week were included, without case selection or exclusion criteria, ensuring a representative sample of routine clinical practice. Visits were performed by certified thoracic surgeons; Six surgeons participated during the study week.

Data collected included patient demographics (age, sex), consultation type (first visit or follow-up), consultation modality (in-person or telemedicine), and the final clinical diagnosis established by the surgeon. The full distribution of first vs. follow-up and in-person vs. telemedicine is reported in Results.

### Artificial intelligence model

Scholar GPT, (OpenAI, version accessed January 2025) a large language model based on GPT-4 architecture, was selected due to its ability to access and synthesize information from biomedical databases, including PubMed and evidence-based clinical guidelines. The model was accessed via a dedicated medical consultation interface during *January* 2025, with output traceability. The system had no connectivity to the hospital EHR and no local fine-tuning with institutional data.

### Procedure and data collection

For each consultation, the attending surgeon documented the primary reason for the visit, relevant clinical history, imaging results, and the diagnostic and therapeutic plan. The same clinical scenario was entered into Scholar GPT, prompting the AI to provide a diagnostic assessment and treatment recommendation. AI responses were recorded verbatim without modifications. The AI received a structured *text* summary (history, comorbidities/medication, and key findings transcribed from imaging reports); no image files were uploaded, and no external oncology notes were provided. Physical examination findings, when available, were included as text.

Additionally, the number of clarification questions generated by the AI before formulating a recommendation was documented to evaluate the model's reasoning process. Across the 81 consultations, the mean number of clarification turns was *1.02*. Two independent thoracic surgeons reviewed the AI output and assigned a concordance score according to the predefined scale.

### Concordance scale and evaluation

A specific 6-point ordinal concordance scale (0–5) was developed to assess the level of agreement between AI-generated recommendations and the surgeons' decisions, focusing on both diagnosis and treatment alignment:

- 0: No diagnosis or treatment proposed.
- 1: Correct diagnosis, no treatment recommendation.
- 2: Correct diagnosis with limited treatment alignment (≤25%).
- 3: Correct diagnosis with partial treatment alignment (≈50%).
- 4: Correct diagnosis with near-complete treatment alignment (≈75%).
- 5: Complete agreement in both diagnosis and treatment.

This scale allowed a detailed evaluation of the AI's clinical performance, distinguishing between correct diagnostic proposals and the adequacy of therapeutic suggestions.

### Statistical analysis

Descriptive statistics were used to summarize the distribution of concordance scores between Scholar GPT's recommendations and the surgeons' decisions. Normality of continuous variables was assessed using the Shapiro–Wilk test. As the concordance scores did not follow a normal distribution (*p* < 0.001), non-parametric tests were employed. Comparisons between groups were performed using the Mann–Whitney *U*-test for two-group comparisons, while categorical variables were assessed with chi-square tests. Additionally, subgroup analyses were performed to compare concordance scores based on diagnosis type, consultation modality (in-person vs. telemedicine), and visit type (first-time vs. follow-up). Statistical significance was set at *p* < 0.05. Given the exploratory scope and one-week sampling, no *a priori* sample-size/power calculation was undertaken. Formal normality testing and inter-rater reliability metrics (e.g., ICC) were not computed; these are acknowledged as limitations and targets for future work.

### Ethical considerations

This study was retrospective and based on anonymized clinical data. All patients had previously signed a general institutional consent authorizing the use of their anonymized data for research purposes. According to institutional policy, no additional ethical approval was required. No patient-identifiable information was entered into the AI system.

## Results

**Figure 1:**
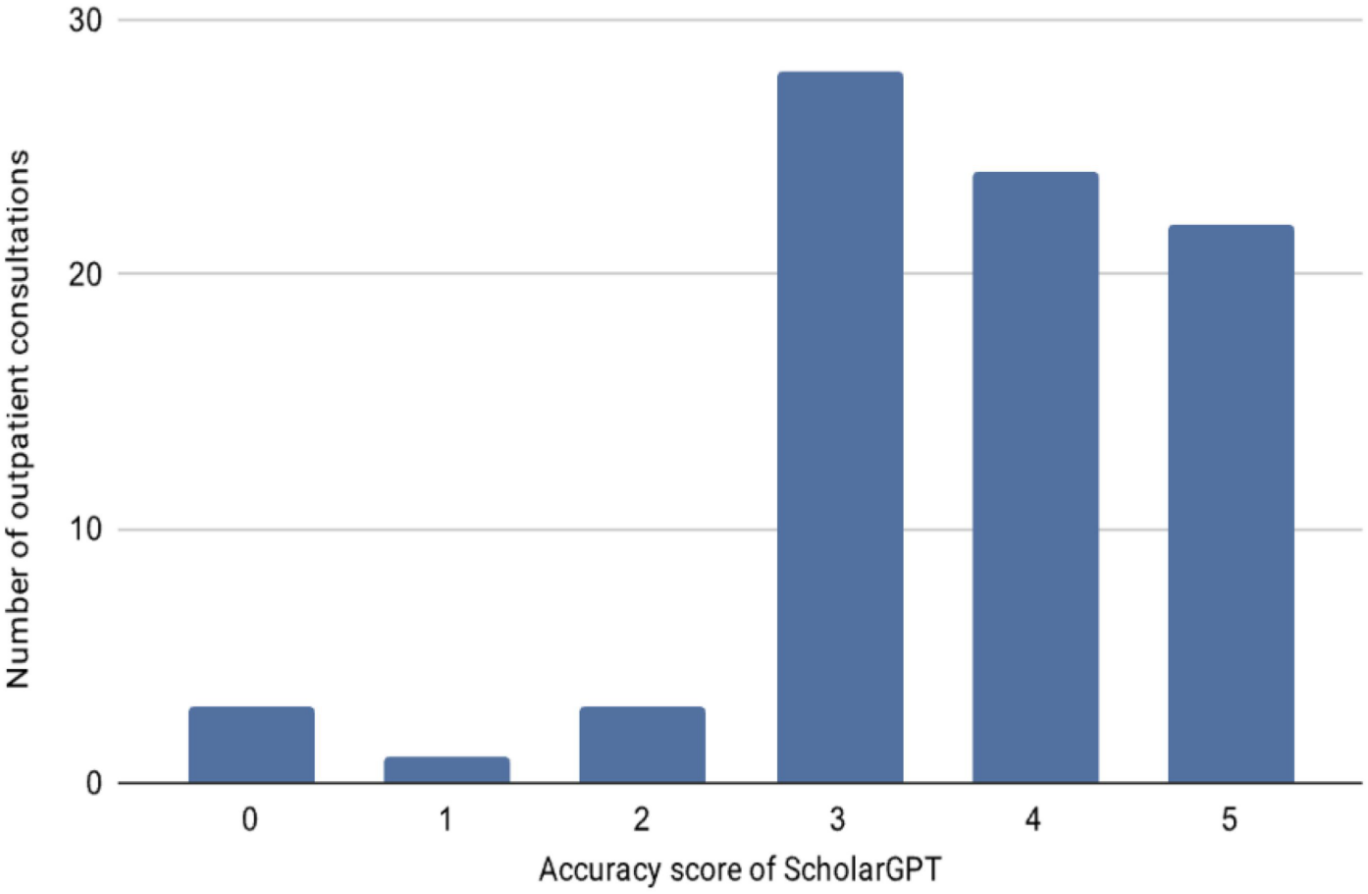
Distribution of concordance scores between Scholar GPT and thoracic surgeons across the 81 outpatient consultations.

A total of 81 thoracic surgery outpatient consultations were analysed to assess the concordance between Scholar GPT's recommendations and the clinical decisions made by thoracic surgeons. The mean concordance score on the 0–5 scale was 3.67 ± 1.17, with individual scores ranging from 0 (no diagnosis or treatment proposed) to 5 (complete agreement in diagnosis and treatment). The distribution of concordance scores is shown in Figure 1.

High concordance (scores 4–5) was observed in 56.8% of cases (*n* = 46), indicating substantial agreement between the AI-generated recommendations and the clinical decisions. Moderate concordance (score 3) occurred in 34.6% of consultations (*n* = 28), while low concordance (scores 0–2) was found in 8.6% of cases (*n* = 7). First visits accounted for 8/81 (9.9%) and follow-up visits for 73/81 (90.1%); in-person consultations were 39/81 (48.1%) and telemedicine 42/81 (51.9%). No statistically significant differences in concordance were observed across visit type (Mann–Whitney *U* = 292.5, *p* = 1.000) or consultation modality (Mann–Whitney *U* = 908.0, *p* = 0.381).

**Figure 2:**
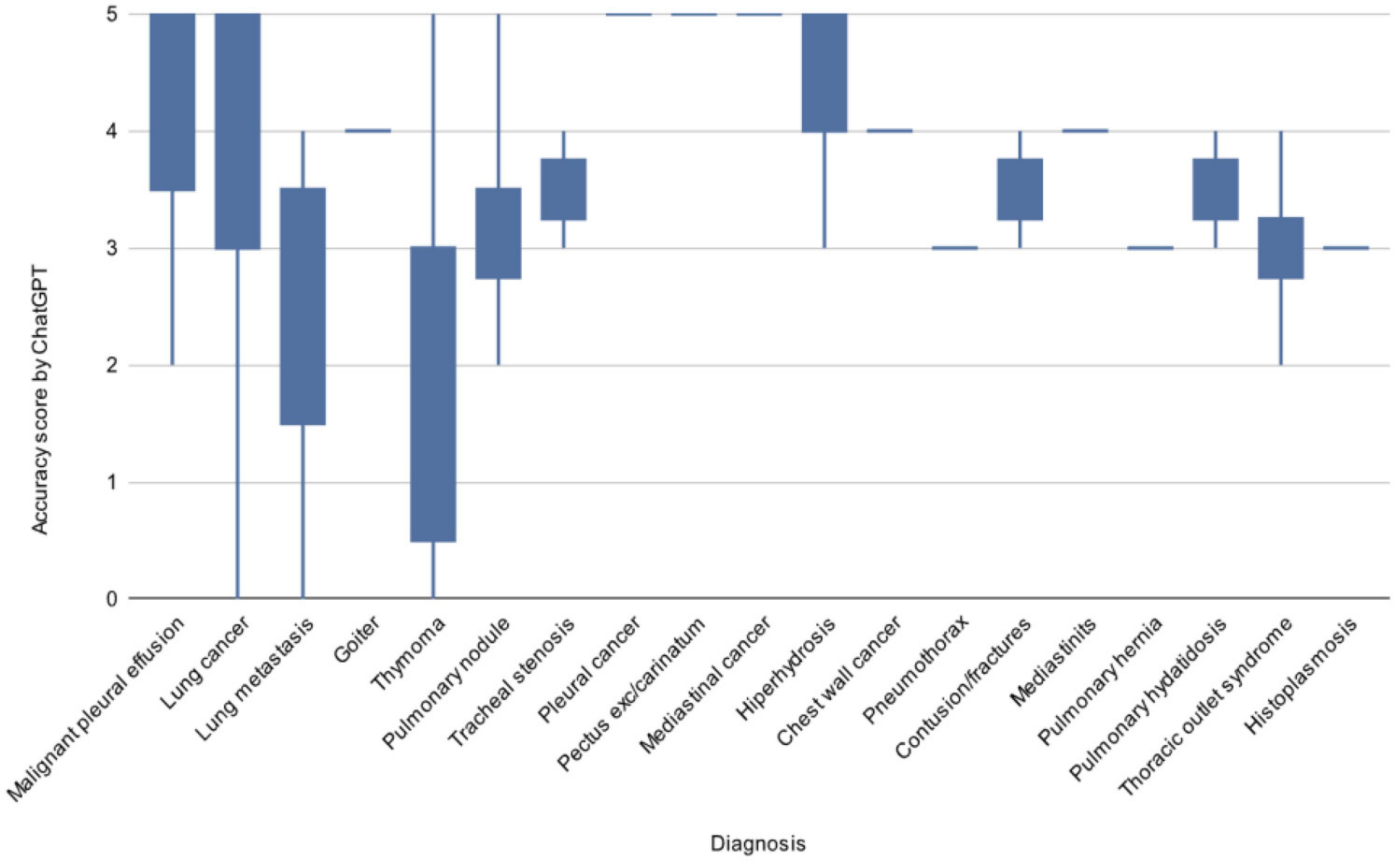
Accuracy scores of Scholar GPT by diagnosis. The plot illustrates higher concordance in oncological cases and greater variability in functional or complex diagnoses.

The AI model performed notably well in oncology-related cases, achieving perfect alignment in diagnoses such as pectus excavatum/carinatum (*n* = 2; mean score 5.0), mediastinal neoplasms (*n* = 1; mean 5.0), and pleural neoplasms (*n* = 1; mean 5.0). In contrast, lower concordance was identified in conditions requiring nuanced clinical judgment or complex decision-making, including: thymoma (*n* = 3; mean 2.0), pulmonary metastases (*n* = 3; mean 2.3), and thoracic outlet syndrome (*n* = 4; mean 3.0). Among primary lung cancer presentations (*n* = 42), the mean concordance was 3.83. The variability in accuracy scores according to diagnosis is represented in Figure 2. Given the small numerators in some subgroups, these estimates should be interpreted cautiously.

Additionally, Scholar GPT demonstrated high efficiency, requiring a mean of 1.02 clarification questions to reach its diagnostic and therapeutic recommendations, with most cases resolved after a single prompt. Typical clarification themes included: (i) staging details (e.g., PET-CT/SUV and nodal stations), (ii) pulmonary function metrics (FEV1, DLCO, and predicted postoperative values), (iii) perioperative risk modifiers (anticoagulation/antiplatelet therapy, frailty/performance status), and (iv) occasional requests for low-yield tests (e.g., tumor markers) in otherwise straightforward scenarios. For instance, in a lung cancer case, the AI asked: “*What are the specific results of the PET-CT, including SUVmax values and any evidence of mediastinal or distant metastasis?*”.

Safety-relevant review did not identify AI recommendations that would be clearly harmful if executed verbatim; however, in low-concordance scenarios some suggestions could plausibly lead to delayed work-up or suboptimal management (e.g., unnecessary testing or premature therapy). These observations reinforce the need for clinician oversight and multidisciplinary team (MDT) review when AI advice diverges from standard pathways.

## Discussion

The integration of large language models (LLMs) into clinical workflows represents a significant advancement in decision support, especially in resource-constrained outpatient settings. Our study explored the use of Scholar GPT as a supportive tool in thoracic surgery consultations and revealed moderate to high concordance between its recommendations and those made by expert clinicians, particularly in oncological cases. These results suggest that LLMs may offer valuable assistance when standard guideline-based decisions are required. This aligns with prior evidence that LLMs perform strongly on knowledge-based tasks and clinical reasoning benchmarks (4, 6) and with observations that foundation models tend to excel on well-specified, structured problems (7).

Recent literature demonstrates the expanding role of AI tools, including LLMs like ChatGPT and Med-PaLM, in improving diagnostic accuracy, triage, and treatment selection across various specialties (5, 9, 10). For instance, AI has shown utility in radiology, pathology, and oncology by offering second-opinion diagnoses and reducing interobserver variability (14). In their study on foundation models for generalist medical AI, Moor et al. reported that such models demonstrate strong performance in standard tasks but often underperform in more nuanced clinical decision-making scenarios (15). However, in surgical fields such as thoracic surgery, real-world validation of LLMs remains scarce.

Our findings align with prior works reporting strong AI performance in well-defined oncological scenarios, where diagnostic and treatment pathways are often governed by standardized guidelines (16, 17). Conversely, lower agreement was observed in functional conditions, metastatic disease, or rare syndromes, echoing previous concerns about AI limitations in complex or ambiguous clinical contexts. Rajpurkar et al. emphasized that although LLMs show high accuracy on benchmark datasets, their reliability in clinical deployment still demands rigorous validation and attention to prompt sensitivity (2).

### Clinical safety implications and the black box problem

A critical aspect that requires thorough discussion, as highlighted by the reviewers, is the clinical safety implications of AI decisions, particularly the potential risks in low-concordance cases. While our safety-relevant review did not identify AI recommendations that would be clearly harmful if executed verbatim, the potential for suboptimal management in low-concordance scenarios represents a significant concern that extends beyond our immediate findings.

Recent comprehensive evaluations have demonstrated that current LLMs are not ready for autonomous clinical decision-making (18). Hager et al. found that LLMs tend to make hasty decisions without consistently following diagnostic guidelines, often omitting essential physical examinations and misinterpreting basic laboratory results—fundamental errors that pose serious risks to patient safety without extensive clinician supervision (18). This finding is particularly relevant to our study, as it suggests that even in cases where we observed moderate concordance, the underlying reasoning process may be fundamentally flawed.

The “black box” nature of LLMs further complicates their integration into surgical decision-making contexts. Unlike traditional clinical decision support tools where the reasoning pathway can be traced and validated, LLMs operate through complex neural networks that make their decision-making process opaque and uninterpretable (19). This lack of transparency poses significant challenges for surgical practice, where understanding the rationale behind a recommendation is crucial for patient safety and medicolegal accountability. As Xu et al. argue, the unexplainability feature of medical AI systems may cause harm that is currently underestimated, particularly when these systems make incomprehensible mistakes that are difficult to detect (19).

The implications of this opacity are particularly concerning in thoracic surgery, where decisions often involve high-stakes interventions with significant morbidity and mortality risks. When an AI system recommends a specific surgical approach or suggests delaying intervention, surgeons need to understand the underlying reasoning to make informed decisions about patient care. The inability to interrogate the AI's decision-making process undermines the fundamental principle of evidence-based medicine and may lead to either inappropriate reliance on AI recommendations or complete rejection of potentially valuable insights.

### Strengths and limitations

This study offers a real-life evaluation of LLM-assisted consultation in a high-volume outpatient service, contributing to a growing body of work emphasizing AI's role in enhancing healthcare efficiency (20). However, important limitations must be noted, and their impact on result interpretation requires careful analysis.

First, LLMs operate without direct access to physical examination findings or dynamic patient interaction, factors essential for nuanced decision-making in surgical practice. This limitation is particularly significant in thoracic surgery, where physical examination findings such as respiratory mechanics, chest wall deformities, and lymph node palpation often provide crucial diagnostic information that cannot be captured in text-based summaries.

Second, the lack of interpretability in LLM outputs—commonly referred to as the “black box” problem—poses a barrier to clinician trust and accountability (21). The absence of baseline intra-group concordance assessment (AI reproducibility and inter-surgeon agreement) as control measures, as noted by the reviewers, represents a significant methodological limitation that prevents us from establishing the reliability of our concordance measurements.

Third, our study lacks formal normality testing before applying *t*-tests and the absence of inter-rater correlation coefficient analysis. These methodological deficiencies impact the interpretation of our results and should be addressed in future studies.

Furthermore, AI outputs may vary based on prompt phrasing, regional practices, and training data, as demonstrated in comparative analyses between generalist LLMs and domain-specific models (22, 23). Although Scholar GPT achieved high scores in our study, its performance cannot be generalized without further multi-centre validation. The small sample size of 81 cases is insufficient for multiple subgroup analyses, resulting in inadequate statistical power for many of our diagnostic-specific comparisons.

A major limitation of this study is the absence of inter-rater reliability assessment. While two independent thoracic surgeons assigned concordance scores, only the final consensus scores were recorded, preventing calculation of inter-rater agreement statistics such as Cohen's kappa or ICC. This represents a significant methodological deficiency that limits confidence in the reliability of our primary outcome measure.

### Applications in thoracic surgery

Despite these limitations, the potential applications of AI in thoracic surgery continue to expand. Recent comprehensive reviews have highlighted AI's growing role in enhancing diagnostic accuracy, surgical precision, and postoperative care in thoracic surgery (24). AI applications now span the entire perioperative period, from preoperative risk assessment and surgical planning to intraoperative guidance and postoperative monitoring (5).

In the preoperative phase, AI has demonstrated promise in imaging analysis, tumor classification, and surgical candidacy assessment. During surgery, AI-assisted augmented reality systems have shown feasibility in robotic lung surgery, achieving high accuracy in gesture recognition and potentially improving surgical precision (25). Postoperatively, AI assists in pathology assessment, complication prediction, and long-term outcome modelling.

However, as noted in recent scoping reviews, significant challenges remain, including the need for larger, more diverse datasets, standardization of AI applications across institutions, and development of robust validation frameworks specific to thoracic surgery (26).

### Implications and future directions

LLMs may serve as cognitive aids, particularly in settings with limited subspecialty coverage, such as remote clinics or after-hours consultations. Yet, AI should complement—not replace—clinical judgment, especially in patient-specific scenarios involving comorbidities or psychosocial factors (27).

Future efforts should focus on fine-tuning LLMs using thoracic surgery-specific datasets, integrating multimodal inputs (e.g., imaging, labs), and enabling real-time feedback loops from clinical users (11). Ethical frameworks and regulatory guidelines will also be essential to ensure responsible AI deployment in patient care (28). Most importantly, future studies must address the methodological limitations identified in our work, including larger sample sizes, multi-centre designs, formal inter-rater reliability assessments, and comprehensive safety evaluations.

The development of explainable AI systems that can provide transparent reasoning for their recommendations will be crucial for gaining clinician trust and ensuring safe integration into surgical practice. Until these challenges are addressed, LLMs should be used with extreme caution in clinical decision-making, with mandatory human oversight and validation of all AI-generated recommendations.

## Conclusion

Large language models, such as Scholar GPT, demonstrate high concordance with thoracic surgeons in outpatient decision-making, particularly in oncological cases. However, its performance was more variable in complex or functional diagnoses, highlighting the indispensable role of human expertise in nuanced clinical scenarios. While AI-based decision-support systems have the potential to enhance clinical efficiency, their integration should focus on augmenting, rather than replacing, expert decision-making. Future advancements should prioritize real-time clinical feedback and multimodal patient data integration to optimize the reliability and applicability of AI-assisted decision-making in thoracic surgery.

## Data Availability

The raw data supporting the conclusions of this article will be made available by the authors, without undue reservation.

## References

[1] Topol EJ. High-performance medicine: the convergence of human and artificial intelligence. Nat Med. (2019) 25:44–56. 10.1038/s41591-018-0300-7 30617339

[2] Rajpurkar P Chen E Banerjee O Topol EJ. AI In health and medicine. Nat Med. (2022) 28:31–8. 10.1038/s41591-021-01614-0 35058619

[3] Yu KH Beam AL Kohane IS. Artificial intelligence in healthcare. Nat Biomed Eng. (2018) 2:719–31. 10.1038/s41551-018-0305-z 31015651

[4] Singhal K Azizi S Tu T Mahdavi SS Wei J Chung HW Large language models encode clinical knowledge. Nature. (2023) 620:172–80. 10.1038/s41586-023-06291-2 37438534 PMC10396962

[5] Bellini V Pinotti E Sozzi M Bignami E. Artificial intelligence in thoracic surgery: a narrative review. J Thorac Dis. (2021) 13:6487–502. 10.21037/jtd-21-761 PMC8743413 35070380

[6] Lee P Bubeck S Petro J. Benefits, limits, and risks of GPT-4 as an AI chatbot for medicine. N Engl J Med. (2023) 388:1233–9. 10.1056/NEJMsr2214184 36988602

[7] Bommasani R Hudson DA Adeli E Altman R Arora S von Arx S On the opportunities and risks of foundation models. arXiv preprint arXiv:2108.07258. (2021).

[8] Thirunavukarasu AJ Ting DSJ Elangovan K Gutierrez L Tan TF Ting DSW. Large language models in medicine. Nat Med. (2023) 29:1930–40. 10.1038/s41591-023-02448-8 37460753

[9] Nori H King N McKinney SM Carignan D Horvitz E. Capabilities of GPT-4 on medical challenge problems. arXiv preprint arXiv:2303.13375. (2023).

[10] Tu T Azizi S Driess D Schaekermann M Amin M Chang PC Towards generalist biomedical AI. N Engl J Med. (2023) 389:1180–9. 10.1056/NEJMra220477837754283

[11] Amann J Blasimme A Vayena E Frey D Madai VI. Explainability for artificial intelligence in healthcare: a multidisciplinary perspective. BMC Med Inform Decis Mak. (2020) 20:310. 10.1186/s12911-020-01332-6 33256715 PMC7706019

[12] Rudin C. Stop explaining black box machine learning models for high stakes decisions and use interpretable models instead. Nat Mach Intell. (2019) 1:206–15. 10.1038/s42256-019-0048-x 35603010 PMC9122117

[13] Ghassemi M Oakden-Rayner L Beam AL. The false hope of current approaches to explainable artificial intelligence in health care. Lancet Digit Health. (2021) 3:e745–50. 10.1016/S2589-7500(21)00208-9 34711379

[14] Brügge E Ricchizzi S Arenbeck M Keller MN Dittrich F Seyfarth S Large language models improve clinical decision making of medical students through patient simulation and structured feedback: a randomized controlled trial. BMC Med Educ. (2024) 24:1399. 10.1186/s12909-024-06399-7 39609823 PMC11605890

[15] Moor M Banerjee O Abad ZSH Krumholz HM Leskovec J Topol EJ Foundation models for generalist medical artificial intelligence. Nature. (2023) 616:259–65. 10.1038/s41586-023-05881-4 37045921

[16] Gaber F Ahmed A Abdelrazek M Grundy J Susilo W. Evaluating large language model workflows in clinical decision support. NPJ Digit Med. (2025) 8:11. 10.1038/s41746-025-01684-1 40346344 PMC12064692

[17] Li J Zhou Z Lyu H Wang Z. Large language models-powered clinical decision support: enhancing or replacing human expertise? Intell Med. (2025) 5:14–20. 10.1016/j.imed.2025.01.002

[18] Hager P Jungmann F Holland R Bhagat K Hubrecht I Knauer M Evaluation and mitigation of the limitations of large language models in clinical decision-making. Nat Med. (2024) 30:2613–22. 10.1038/s41591-024-03097-1 38965432 PMC11405275

[19] Xu H Shuttleworth KMJ. Medical artificial intelligence and the black box problem: a view based on the ethical principle of “do no harm”. Intell Med. (2024) 4:77–85. 10.1016/j.imed.2023.08.001

[20] Lammert J Ravi N Rajpurkar P. Expert-guided large language models for clinical decision support. JAMA Netw Open. (2024) 7:e2439297. 10.1001/jamanetworkopen.2024.39297

[21] Winkler PW Ravi N Rajpurkar P. Risks, limitations, safety and verification of medical AI systems. Nat Med. (2025) 31:45–52. 10.1038/s41591-024-03298-839833407

[22] Toal M Hill C Quinn M O'Neill C Molloy EJ Denton E Large language Models’ clinical decision-making on when to perform a kidney biopsy: comparative study. J Med Internet Res. (2025) 27:e73603. 10.2196/73603 40966592 PMC12445783

[23] McCoy LG Nagaraj S Morgado F Harish V Das S Celi LA. What do medical students think of AI? Implications for curricula and ethics. Acad Med. (2020) 95:1858–64. 10.1097/ACM.0000000000003537

[24] Leivaditis V Maniatopoulos AA Lausberg H Koletsis E Prokakis C Koletsis N Artificial intelligence in thoracic surgery: a review bridging innovation and clinical practice for the next generation of surgical care. J Clin Med. (2025) 14:2729. 10.3390/jcm14082729 40283559 PMC12027631

[25] Sadeghi AH Bakhuis W van der Sande L Goos T Goense L Akbari Aghdam K Artificial intelligence-assisted augmented reality robotic lung surgery: navigating the future of thoracic surgery. JTCVS Tech. (2024) 26:121–5. 10.1016/j.xjtc.2024.05.003 39156519 PMC11329169

[26] Seastedt KP Moukheiber D Muniappan A Gaissert HA Wright CD Lanuti M A scoping review of artificial intelligence applications in thoracic surgery. Eur J Cardiothorac Surg. (2022) 61:239–47. 10.1093/ejcts/ezab422 34601587 PMC8932394

[27] Abbaker N Minervini F Guttadauro A Solli P Cioffi U. The future of artificial intelligence in thoracic surgery for non-small cell lung cancer treatment: a narrative review. Front Oncol. (2024) 14:1347464. 10.3389/fonc.2024.1347464 38414748 PMC10897973

[28] Ong JCL Bharath A Sharma A Kulkarni S Subramanian V Garg T Ethical and regulatory challenges of large language models in medicine. Lancet Digit Health. (2024) 6:e366–8. 10.1016/S2589-7500(24)00061-X 38658283

